# Paired vagus nerve stimulation for post-stroke shoulder motor recovery: corticospinal–C5 motor network plasticity and rehabilitation applications

**DOI:** 10.3389/fneur.2026.1949342

**Published:** 2026-09-03

**Authors:** Xiang Ji, Weiran Zheng, Wei Ma, Jiaying Liu, Guizhi Xu

**Affiliations:** 1 School of Electrical Engineering, Hebei University of Technology, Tianjin, China; 2 State Key Laboratory of Smart Power Distribution Equipment and System, Hebei University of Technology, Tianjin, China; 3 School of Health Sciences and Biomedical Engineering, Hebei University of Technology, Tianjin, China

**Keywords:** C5 motor network, corticospinal tract, neuroplasticity, neurorehabilitation, paired vagus nerve stimulation, shoulder motor recovery, stroke

## Abstract

Shoulder motor impairment after stroke restricts reaching, self-care, balance reactions, and the use of mobility aids. Paired vagus nerve stimulation (pVNS), in which brief cervical vagus nerve stimulation is temporally coupled to task-specific rehabilitation, improves overall upper-extremity impairment and activity in selected people with chronic ischemic stroke. Its shoulder-specific effects, however, remain uncertain. This Mini Review evaluates pVNS through a corticospinal–C5 motor-network frameworkthat links movement-related cortical ensembles, descending corticospinal and reticulospinal pathways, cervical C5 motor pools, and the peripheral motor units controlling the supraspinatus, deltoid, infraspinatus, and scapular stabilizers. Preclinical studies show that appropriately timed pVNS recruits noradrenergic, cholinergic, and serotonergic systems, promotes movement-specific motor-cortical plasticity, and augments forelimb recovery after experimental stroke. Randomized clinical trials demonstrate durable improvement in composite upper-limb outcomes, leading to regulatory approval for moderate-to-severe arm impairment after chronic ischemic stroke. Nevertheless, secondary analyses indicate a clearer treatment advantage for distal than proximal components, and shoulder abduction has not been tested as an independent primary endpoint. We therefore outline a candidate shoulder-focused rehabilitation model combining graded antigravity abduction, external rotation, scapular control, and functional reaching with defined stimulation pairing and multimodal monitoring. The model presupposes an intact peripheral C5 motor unit: pVNS cannot repair complete C5-root, upper-trunk, suprascapular, or axillary nerve denervation. Shoulder-specific trials incorporating kinematics, electromyography, transcranial magnetic stimulation, and tract imaging are required before efficacy for proximal recovery can be claimed.

## 1 Introduction

Shoulder motor recovery is a clinically meaningful gateway to arm use after stroke. Early shoulder abduction, particularly when combined with finger extension, predicts later upper-limb capacity, while active shoulder range contributes strongly to functional performance (1, 2). Persistent weakness also interacts with pain, subluxation, soft-tissue injury, abnormal tone, and learned non-use; shoulder pain remains common and can further restrict practice (3). Restoring proximal control is therefore not merely a matter of increasing a joint angle. It establishes the mobile but stable base required for reaching, hand positioning, protective reactions, and many activities of daily living.

Paired vagus nerve stimulation (pVNS) is a targeted-plasticity approach in which a brief train of left cervical vagus nerve stimulation is delivered during repeated, task-specific upper-limb practice. Pilot trials and the pivotal VNS-REHAB study reported greater improvement in composite upper-extremity impairment and function than rehabilitation paired with sham stimulation (4–6). In 2021, the US Food and Drug Administration approved the implanted system for use during rehabilitation to reduce upper-extremity motor deficits in adults with chronic ischemic stroke and moderate-to-severe arm impairment (7).

The regulatory indication and trials concern the upper extremity as a whole, not isolated shoulder recovery. Moreover, a stroke-related central paresis is biologically different from traumatic or non-traumatic denervation of the C5 root or its branches. This distinction is central to the present review. We use the term “corticospinal–C5 motor network” solely as a translational, hypothesis-generating framework, and not as a formally defined anatomical or physiological entity, to describe the pathway from cortical motor commands through descending systems and cervical C5 circuitry to the peripheral motor units that generate shoulder abduction and external rotation. We examine the mechanistic rationale, clinical evidence, rehabilitation applications, and evidence gaps for using pVNS to improve post-stroke shoulder motor function while defining when peripheral C5 pathology makes this strategy insufficient.

## 2 Shoulder motor recovery as a corticospinal–C5 system problem

### 2.1 Proximal control, weakness, and abnormal synergies

Normal shoulder elevation depends on coordinated activation of the supraspinatus and deltoid, external-rotation control from the infraspinatus and teres minor, and scapular upward rotation and posterior tilt. After stroke, loss of selective descending drive is compounded by stereotyped coactivation. Hemiparetic participants generate abnormal shoulder–elbow torque combinations, and increasing shoulder-abduction loading can reduce reachable workspace by strengthening the expression of a flexion synergy (8–10). Progressive abduction loading can nevertheless be therapeutic when introduced within a task-oriented program that challenges rather than reinforces the synergy (11).

Compensation further complicates interpretation. Trunk displacement and scapular hiking may increase endpoint reach without restoring glenohumeral control (12). Muscle-synergy analyses show that post-stroke motor patterns can reflect merging or fractionation of pre-existing modules, consistent with altered cortical control rather than a single weak muscle (13). Recruitment of contralesional cortico-reticulospinal pathways may help generate gross force but can also couple shoulder abduction to involuntary elbow and wrist flexion, particularly at higher effort (14). Thus, a shoulder intervention should be judged by movement quality, load tolerance, and selective recruitment—not only by task completion.

### 2.2 The corticospinal–C5 motor-network framework

Within this framework, residual ipsilesional corticospinal output provides relatively fractionated control, whereas reticulospinal and propriospinal systems contribute posture, multijoint coordination, and compensatory drive. These descending signals converge on cervical interneurons and C5-dominant motor pools. The peripheral output then travels principally through the suprascapular and axillary nerves to the supraspinatus, infraspinatus, and deltoid, with additional C5–C7 contributions to scapular stabilizers. The clinically observed movement is therefore the product of a serial system: cortical selection, descending transmission, spinal integration, peripheral conduction, neuromuscular transmission, muscle force, and joint mechanics.

Corticospinal tract integrity is a major determinant of upper-limb recovery and can improve prognostic stratification beyond bedside severity alone (15, 16). Standard outcomes such as the Fugl–Meyer Assessment–Upper Extremity (FMA-UE) and Wolf Motor Function Test capture complementary impairment and activity domains but do not by themselves localize failure within this serial system (17, 18). A low proximal score may arise from central weakness, flexion synergy, pain, contracture, rotator-cuff pathology, or an unrecognized C5-root, upper-trunk, suprascapular, or axillary neuropathy.

This distinction defines the biological ceiling of pVNS. The therapy may reinforce residual central commands and improve recruitment of an anatomically intact C5 motor unit. It cannot reconnect an avulsed root, regenerate a transected terminal nerve, or directly reinnervate a completely denervated muscle. When weakness is disproportionate, sensory loss overlies the lateral shoulder, tendon reflexes are depressed, or atrophy and fasciculation are present, electrodiagnostic testing and targeted shoulder or plexus imaging should precede a central-neuromodulation interpretation.

## 3 Why pairing VNS with shoulder practice may promote plasticity

### 3.1 Temporally gated neuromodulation

Vagal afferents project to the nucleus tractus solitarius and engage brainstem and basal-forebrain neuromodulatory systems. The therapeutic concept is not that VNS directly activates the paretic shoulder. Rather, a brief stimulation train creates a transient plasticity-permissive state while the neural ensemble responsible for a practiced movement is active. Repeated pairing can therefore reinforce task-relevant circuits more selectively than continuous stimulation.

In intact rats, pairing VNS with specific distal or proximal forelimb movements reorganized the corresponding primary motor-cortex representations, producing movement-specific motor maps (19). Experimental stroke studies subsequently showed greater recovery of forelimb strength or task performance with VNS paired to rehabilitation than with rehabilitation alone, unpaired stimulation, or delayed stimulation (20, 21). The timing and amount of stimulation influence recovery, supporting the importance of temporal contingency (22). Benefits have also been reported after experimental intracerebral hemorrhage, in chronic ischemic injury, and in aged animals, indicating that the effect is not limited to a single lesion model or young subjects (23–25). Long-term retention and generalization beyond the trained task have been demonstrated, although the extent of generalization depends on the trained behavior and measurement context (26).

### 3.2 Neuromodulatory and circuit mechanisms

The plasticity response is parameter dependent. Moderate stimulation intensities can produce greater motor-map reorganization than lower or higher intensities, emphasizing that more current is not necessarily more effective (27). Cholinergic integrity is required for VNS-driven motor-cortical reorganization (28). VNS also evokes rapid locus-coeruleus activity, while noradrenergic and serotonergic signaling is necessary for directed cortical plasticity (29, 30). Local α2-adrenergic receptor activation within motor cortex contributes to this effect (31). More recent circuit-level work indicates that VNS can selectively modulate behaviorally engaged cortical neurons through cholinergic reinforcement rather than causing nonspecific global learning enhancement (32).

**Figure 1:**
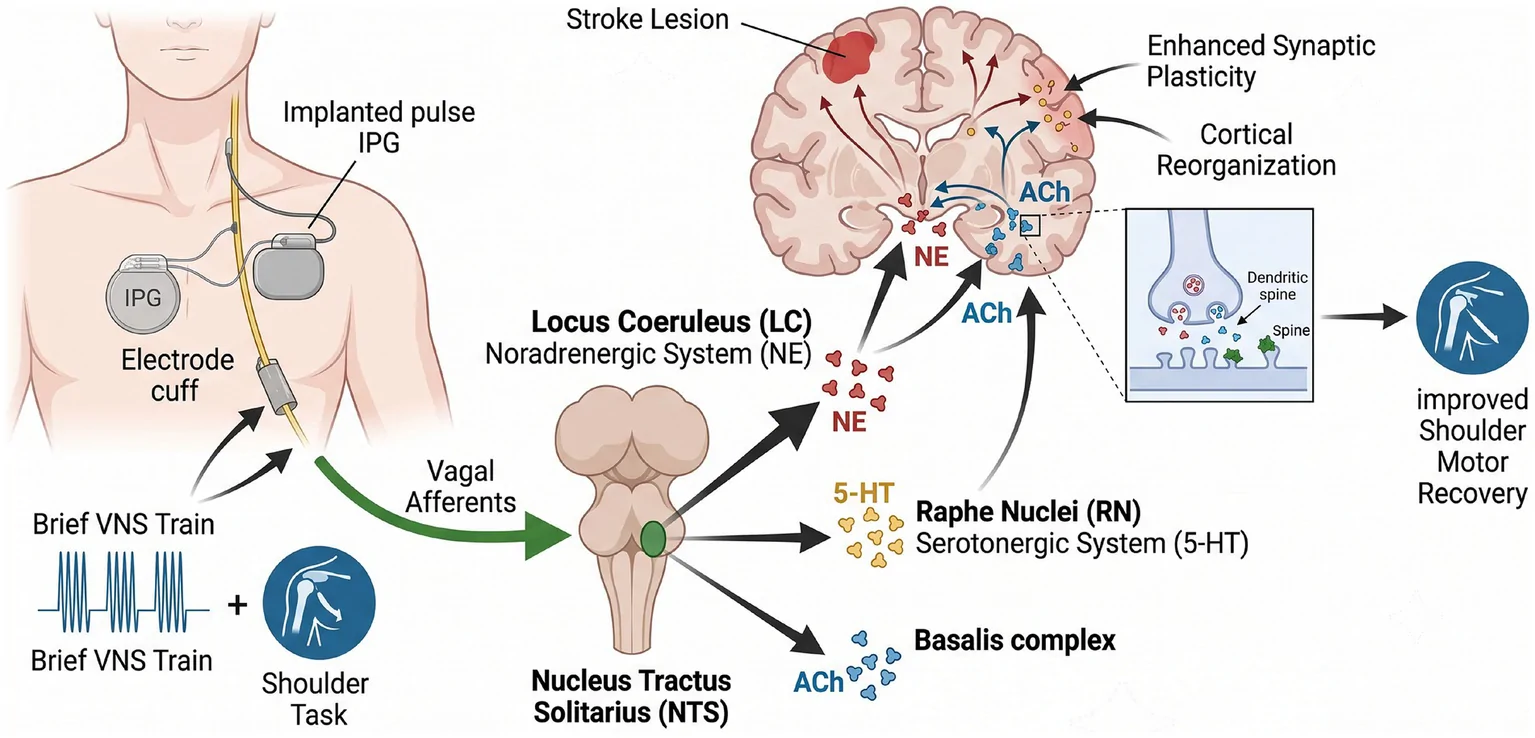
Proposed mechanism by which paired vagus nerve stimulation may facilitate shoulder motor recovery after stroke. A brief VNS train delivered with a defined shoulder task activates vagal afferents and the Nucleus Tractus Solitarius, engaging noradrenergic, cholinergic, and serotonergic neuromodulatory systems. ACh, acetylcholine; AXN, axillary nerve; LC, Locus Coeruleus; M1, Primary Motor Cortex; NE, norepinephrine; NTS, Nucleus Tractus Solitarius; pVNS, paired vagus nerve stimulation; SSN, suprascapular nerve; 5-HT, serotonin.

For shoulder recovery, these findings support a testable sequence: stimulation aligned with a well-defined shoulder task engages phasic neuromodulation; task-active motor-cortical ensembles are preferentially reinforced; residual corticospinal transmission becomes more effective or better coordinated; and cervical C5 motor pools recruit the appropriate muscles with less maladaptive multijoint coupling. Direct evidence for every link in humans is not yet available. In particular, no study has shown that pVNS selectively strengthens corticospinal projections to C5 shoulder motor pools or suppresses pathological reticulospinal synergy. The sequence should therefore be regarded as a mechanistic model for trial design, not as an established clinical mechanism (Figure 1).

### 3.3 Why a shoulder-specific pairing strategy may matter

Shoulder tasks differ from hand tasks in biomechanical demand and neural control. Antigravity abduction increases effort and can expose flexion synergy, whereas externally supported reaching permits more selective practice. A rational progression would begin with pain-free scapular-plane movement under gravity compensation, then add external rotation, progressive abduction loading, multidirectional reach, and object transport. Stimulation should be paired with the intended task phase—such as initiation of selective abduction, controlled transition into reach, or successful placement—rather than delivered indiscriminately during passive movement.

This proposal follows the movement-specificity of preclinical plasticity, but it has not been validated clinically. The optimal definition of a “successful” proximal repetition is also unknown. Kinematic thresholds, therapist judgment, surface electromyography, or wearable sensors could trigger stimulation, yet each method emphasizes a different target: endpoint success, movement quality, selective muscle activation, or dose. Movement onset, peak abduction, and successful target acquisition should therefore be regarded as candidate triggers for prospective comparison hypotheses for future protocol development rather than established, evidence-based clinical recommendations. The optimal proximal stimulation trigger remains unknown. Future protocols should report not only the number of stimulations but also which movement phase and motor solution were reinforced.

## 4 Clinical evidence and the shoulder-specific evidence boundary

The first randomized clinical pilot enrolled 21 participants with chronic ischemic stroke and moderate-to-severe upper-limb impairment; pVNS was feasible and acceptably safe, and the per-protocol analysis suggested a larger FMA-UE gain than rehabilitation alone (4). A subsequent blinded, sham-controlled pilot in 17 implanted participants found a day-90 mean FMA-UE improvement of 9.5 points with active pVNS and 3.8 points with sham pairing; 88% versus 33% achieved the prespecified clinically meaningful response (5). Exploratory pooled imaging and clinical analyses did not identify a robust baseline predictor of treatment-specific response, partly because of small samples (33). One-year follow-up of the second pilot showed sustained pooled gains and feasible home use (34).

The pivotal VNS-REHAB trial randomized 108 participants at least 9 months after ischemic stroke. Both groups received 18 in-clinic sessions over 6 weeks followed by home exercise; active stimulation consisted of 0.8 mA, 100-μs pulses at 30 Hz for 0.5 s. The day-1 FMA-UE gain was 5.0 points with pVNS and 2.4 points with sham, and the 90-day clinically meaningful response rate was 47% versus 24%. Functional gains on the Wolf Motor Function Test also favored pVNS (6). *Post hoc* subgroup analyses found no clear interaction with age, sex, baseline severity, time since stroke, side, or cortical involvement within the enrolled population (35). Pilot follow-up suggested persistence at 2 and 3 years (36), and later pivotal-trial follow-up reported durable improvement across impairment, activity, and quality-of-life measures among available participants (37). Neuroimaging and clinical analyses suggest that treatment response is not explained by a single simple lesion metric, reinforcing the need for multimodal prediction (38, 39).

The central limitation for the present topic is segment specificity. A component analysis of VNS-REHAB found significant active-versus-sham advantages for wrist and hand components but not for the proximal FMA-UE component (40). A separate secondary proximal–distal analysis of the same VNS-REHAB trial population, using a different analytical approach, likewise concluded that distal impairment improved more with pVNS, while proximal gains were similar between groups (41). Both analyses are secondary, draw on the same trial cohort, and were not designed around shoulder abduction biomechanics, but they prevent a claim that pVNS has already demonstrated a shoulder-specific treatment effect. At present, the evidence supports pVNS for composite upper-extremity recovery; its incremental value for the shoulder remains an open question.

## 5 Rehabilitation applications for shoulder motor recovery

### 5.1 Patient selection and diagnostic safeguards

Current evidence most directly applies to adults with chronic ischemic stroke, moderate-to-severe arm impairment, sufficient cognition and communication to participate, and enough residual movement to practice repeated tasks. Selection for a shoulder-focused protocol should add a proximal phenotype: limited active abduction or external rotation that is not fully explained by pain, fixed contracture, severe glenohumeral pathology, or complete peripheral denervation.

A structured examination should document active and passive range, scapulohumeral rhythm, subluxation, pain behavior, tone, reflexes, sensation over the C5 and axillary territories, and selective activation of the supraspinatus, deltoid, infraspinatus, serratus anterior, and trapezius. Marked atrophy, depressed biceps reflex, lateral-shoulder sensory loss, or weakness extending beyond the expected central pattern should prompt nerve-conduction studies, needle electromyography, and appropriate cervical, plexus, or shoulder imaging. pVNS should not be used to delay repair or reconstruction of a surgically remediable C5-root or peripheral-nerve lesion.

Because hemiplegic shoulder dysfunction after stroke is characteristically multifactorial the dominant aetiology should be delineated before targeted treatment (42), and assessment conventionally characterises the deficit as central or peripheral and focal or diffuse while grading tone and subluxation (43). For pVNS candidacy we therefore combine the structured examination described above with active screening for coexisting peripheral involvement: focal or disproportionate weakness, lateral upper-arm sensory loss in the axillary or C5 territory, a depressed or asymmetric biceps or brachioradialis reflex, focal atrophy, or fasciculation. The axillary nerve deserves particular scrutiny, being the nerve most frequently injured after glenohumeral dislocation, so proximal weakness following a fall or dislocation warrants specific attention (44). Where any such feature is present or localisation remains uncertain, targeted electrodiagnostic and imaging evaluation is indicated: nerve conduction studies with needle electromyography, ideally performed no sooner than three to 4 weeks after onset so that denervation changes have had time to appear (45); cervical-spine MRI for radiculopathy or nerve-root pathology; brachial-plexus MRI or magnetic resonance neurography, which improves depiction of plexus and branch-nerve lesions that conventional sequences may miss (46); and shoulder ultrasound or MRI where rotator-cuff, dislocation, or capsular disease is suspected. pVNS should proceed only after multidisciplinary confirmation that the peripheral motor unit is viable, that implant risk is acceptable, and that treatment will not delay repair of a surgically remediable lesion. Implant candidacy also requires review of surgical, cardiac, respiratory, swallowing, and voice-related risks and device-specific contraindications. Post-implant monitoring should include wound status, hoarseness, cough, throat discomfort, dysphagia, pain, and tolerance of stimulation. These responsibilities are best shared across neurology, rehabilitation medicine, surgery, therapy, and nursing rather than assigned to the stimulation device alone.

### 5.2 Task design and dose

The rehabilitation task is the informational content of pVNS. A shoulder-focused program should prioritize high-quality, high-repetition actions that are meaningful and progressively loaded. Early tasks may include supported scapular-plane abduction, controlled external rotation with the arm at the side, and low-friction forward reach. Later tasks can add antigravity elevation, reaching to shelves at graded heights, hand-to-head actions, object transport, and bimanual activities. Progressive shoulder-abduction loading should be sufficient to challenge descending control but low enough to avoid repeated collapse into flexion synergy or trunk compensation (9–11).

Therapists should define the event that triggers each stimulation. Triggering at movement onset may reinforce initiation; triggering near peak abduction may reinforce antigravity control; triggering after accurate target acquisition may reinforce successful coordination. A closed-loop system could use inertial sensors and electromyography to detect both angle and muscle selectivity. Until such strategies are tested, the clinical protocol should adhere to approved stimulation parameters and established device training rather than extrapolating experimental intensity or frequency changes.

Home practice is important for dose accumulation, but unsupervised repetitions may reinforce compensation. Remote review, wearable feedback, caregiver education, and nursing follow-up can support adherence and identify pain, fatigue, device intolerance, or functional decline. The goal is not maximal repetition at any cost; it is repeated pairing of stimulation with the desired motor solution.

Table 1 organizes the parameters that a shoulder-focused pVNS protocol would need to prespecify and report, together with the rationale or safeguard for each and the corresponding evidence gap. Because shoulder-specific loading, dose, and trigger thresholds have not been validated, the table states what should be reported rather than presenting fixed values as established standards; this follows established guidance on complete intervention reporting (47) and on quantifying rehabilitation dose as delivered amount rather than scheduled time (48).

**Table 1:** Parameters that a shoulder-focused paired vagus nerve stimulation protocol should prespecify and report, with rationale and current evidence gaps.

Domain	What a trial should prespecify and report	Rationale or safeguard	Unresolved evidence gap
Abduction loading and progression	Starting plane and support condition; level of gravity compensation; absolute or body-weight-normalized abduction load; explicit criteria for progressing or regressing load.	Progressive abduction loading challenges descending control, but excessive load elicits flexion synergy and reduces reachable workspace (9–11).	No validated load threshold marks the point at which pairing stops reinforcing selective control and begins reinforcing synergy.
Task selection and sequence	Ordered task list from supported scapular-plane abduction and external rotation through antigravity elevation, graded-height reaching, and object transport.	Preclinical plasticity is movement-specific, so the paired task defines what is reinforced (19).	Whether proximal task sets produce shoulder-specific cortical reorganization in humans is untested.
Supervised dose	Sessions per week, session duration, repetitions per session and per task, and the number of stimulations actually delivered.	Dose–response relationships in stroke rehabilitation depend on quantified amount rather than scheduled time (48); VNS-REHAB used 18 in-clinic sessions over 6 weeks (6).	No shoulder-specific dose has been derived, and the effective proximal repetition count is unknown.
Stimulation trigger	The exact event that opens the stimulation window (movement onset, peak abduction, or verified target acquisition) and the method used to detect it.	Temporal contingency between stimulation and the active movement determines whether plasticity is directed (19, 22, 32).	Candidate triggers have never been compared prospectively; the optimal proximal trigger is unknown.
Movement-quality gates	Predefined kinematic or electromyographic criteria that disqualify a repetition, such as trunk displacement, scapular hiking, or elbow and wrist flexor coupling.	Endpoint success can be achieved by compensation rather than restored glenohumeral control (12, 14).	No consensus definition of a successful proximal repetition exists.
Home training	Prescribed home tasks, target repetitions, frequency of supervision or remote review, and the method used to capture adherence.	Home practice accumulates dose, but unsupervised repetition can consolidate compensatory patterns.	The safe ratio of supervised to unsupervised proximal practice is undefined.
Electrical settings and safety	Device parameters as approved (0.8 mA, 100 μs pulses at 30 Hz for 0.5 s in VNS-REHAB), together with monitoring of hoarseness, cough, dysphagia, and wound status.	Motor-cortical plasticity is intensity-dependent and non-monotonic, so parameters should not be extrapolated from experimental work (6, 27).	Whether proximal training benefits from parameters different from those used for distal training is untested.
Outcome and fidelity measures	FMA-UE proximal items and the Wolf Motor Function Test alongside prespecified shoulder endpoints, plus planned versus delivered intervention.	Retains continuity with existing pVNS evidence (17, 18); complete intervention reporting enables replication (47).	Shoulder-specific endpoints have not been validated as primary outcomes in pVNS trials.

### 5.3 Outcomes and biomarkers

A shoulder-focused trial should retain validated global outcomes while adding proximal measures. The FMA-UE shoulder/elbow/forearm items and Wolf Motor Function Test provide continuity with existing pVNS evidence (17, 18). Prespecified shoulder endpoints should include active abduction and external-rotation range, isometric torque, ability to sustain elevation, and patient-reported difficulty with dressing, grooming, reaching, transfers, and mobility-aid use. Three-dimensional kinematics can quantify scapular hiking, trunk displacement, humeral external rotation, and reachable workspace under graded abduction loads.

Surface or intramuscular electromyography can assess selective recruitment and abnormal coupling among deltoid, supraspinatus, biceps, pectoralis, and wrist flexors. Transcranial magnetic stimulation may characterize corticospinal excitability and motor-map changes, while diffusion imaging can estimate corticospinal structural reserve. These biomarkers should be treated as mechanistic intermediates rather than substitutes for useful movement. Serial electrodiagnosis is specifically indicated when a concomitant peripheral C5 lesion is suspected, because central plasticity biomarkers cannot establish peripheral axonal continuity.

## 6 Research gaps and priorities

The first priority is a prospectively registered trial in which shoulder motor recovery is a prespecified target rather than a *post hoc* component. Participants should be stratified by baseline proximal impairment, corticospinal tract integrity, flexion-synergy severity, pain and joint pathology, and peripheral C5 motor-unit status. The intervention should compare a shoulder-enriched pVNS program with dose-matched shoulder rehabilitation and should report task-level stimulation timing.

Second, mechanistic studies should test the proposed corticospinal–C5 pathway directly. Concurrent kinematics and electromyography can determine whether gains reflect true selective control or compensation. Transcranial magnetic stimulation, tract imaging, and possibly spinal neurophysiology can assess whether changes occur in corticospinal recruitment, interhemispheric balance, or descending synergy. Third, adaptive triggering should be evaluated against manual therapist triggering. A sensor-based system could withhold stimulation when a repetition is dominated by trunk lean or flexor coupling, thereby preventing reinforcement of an undesirable solution.

Finally, invasive cervical pVNS should not be assumed equivalent to transcutaneous auricular or cervical stimulation; waveform, fiber recruitment, dose, and evidence base differ. Studies in subacute stroke, hemorrhagic stroke, very severe paresis, and patients with mixed central and peripheral injury remain exploratory. Safety, access, cost, maintenance, and equitable referral require pragmatic evaluation alongside efficacy.

## 7 Discussion

Paired VNS has established a clinically credible principle: a brief neuromodulatory signal can be coupled to rehabilitation to enhance recovery even in chronic stroke. Preclinical work provides a coherent basis in movement-specific motor-cortical plasticity and phasic noradrenergic, cholinergic, and serotonergic reinforcement, while randomized trials support durable improvement in composite upper-limb outcomes. These strengths justify testing a more explicitly proximal rehabilitation strategy.

The evidence boundary is equally important. Shoulder abduction has not been an independent primary endpoint, and available component analyses show a clearer incremental benefit distally than proximally. It is worth separating these positions explicitly. What is established is durable improvement in composite upper-extremity impairment and activity in the trial population (6, 37). What has not been demonstrated is a shoulder-specific treatment effect; the available proximal secondary findings are neutral rather than supportive (40, 41), and no proximal trigger, loading progression, or dosing rule has been validated. The corticospinal–C5 motor-network model therefore organizes hypotheses; it does not establish that pVNS restores C5 motor-pool output in humans. Most importantly, central neuromodulation requires a viable peripheral motor unit. It should complement—not replace—diagnosis and treatment of C5-root, upper-trunk, suprascapular, or axillary nerve injury.

A rigorous next step is not to broaden claims from total FMA-UE scores, but to redesign pairing around shoulder biomechanics and measure the full central-to-peripheral chain. If shoulder-specific efficacy is demonstrated, pVNS could become a targeted non-pharmacologic adjunct for selected patients whose proximal weakness reflects impaired descending control. Until then, its use for shoulder recovery should remain evidence-informed, anatomically screened, and explicitly investigational beyond the approved upper-extremity indication.

## References

[1] Nijland RHM van Wegen EEH Harmeling-van der Wel BC Kwakkel G EPOS Investigators. Presence of finger extension and shoulder abduction within 72 hours after stroke predicts functional recovery: early prediction of functional outcome after stroke: the EPOS cohort study. Stroke. (2010) 41:745–50. doi: 10.1161/STROKEAHA.109.572065 20167916

[2] Beebe JA Lang CE. Active range of motion predicts upper extremity function 3 months after stroke. Stroke. (2009) 40:1772–9. doi: 10.1161/STROKEAHA.108.536763, 19265051 PMC2718540

[3] Lindgren I Jönsson AC Norrving B Lindgren A. Shoulder pain after stroke: a prospective population-based study. Stroke. (2007) 38:343–8. doi: 10.1161/01.STR.0000254598.16739.4e, 17185637

[4] Dawson J Pierce D Dixit A Kimberley TJ Robertson M Tarver B . Safety, feasibility, and efficacy of vagus nerve stimulation paired with upper-limb rehabilitation after ischemic stroke. Stroke. (2016) 47:143–50. doi: 10.1161/STROKEAHA.115.010477, 26645257 PMC4689175

[5] Kimberley TJ Pierce D Prudente CN Francisco GE Yozbatiran N Smith P . Vagus nerve stimulation paired with upper limb rehabilitation after chronic stroke: a blinded randomized pilot study. Stroke. (2018) 49:2789–92. doi: 10.1161/STROKEAHA.118.022279, 30355189

[6] Dawson J Liu CY Francisco GE Cramer SC Wolf SL Dixit A . Vagus nerve stimulation paired with rehabilitation for upper limb motor function after ischaemic stroke (VNS-REHAB): a randomised, blinded, pivotal, device trial. Lancet. (2021) 397:1545–53. doi: 10.1016/S0140-6736(21)00475-X, 33894832 PMC8862193

[7] U.S. Food and Drug Administration. Premarket approval P210007: MicroTransponder Vivistim paired VNS system. (2021). Decision date: August 27, 2021. Available online at: https://www.accessdata.fda.gov/scripts/cdrh/cfdocs/cfpma/pma.cfm?id=P210007 (Accessed July 26, 2026).

[8] Dewald JPA Pope PS Given JD Buchanan TS Rymer WZ. Abnormal muscle coactivation patterns during isometric torque generation at the elbow and shoulder in hemiparetic subjects. Brain. (1995) 118:495–510. doi: 10.1093/brain/118.2.495, 7735890

[9] Sukal TM Ellis MD Dewald JPA. Shoulder abduction-induced reductions in reaching work area following hemiparetic stroke: neuroscientific implications. Exp Brain Res. (2007) 183:215–23. doi: 10.1007/s00221-007-1029-6, 17634933 PMC2827935

[10] Beer RF Ellis MD Holubar BG Dewald JPA. Impact of gravity loading on post-stroke reaching and its relationship to weakness. Muscle Nerve. (2007) 36:242–50. doi: 10.1002/mus.20817, 17486581 PMC2866301

[11] Ellis MD Sukal-Moulton T Dewald JPA. Progressive shoulder abduction loading is a crucial element of arm rehabilitation in chronic stroke. Neurorehabil Neural Repair. (2009) 23:862–9. doi: 10.1177/1545968309332927, 19454622 PMC2833097

[12] Cirstea MC Levin MF. Compensatory strategies for reaching in stroke. Brain. (2000) 123:940–53. doi: 10.1093/brain/123.5.940, 10775539

[13] Cheung VCK Turolla A Agostini M Silvoni S Bennis C Kasi P . Muscle synergy patterns as physiological markers of motor cortical damage. Proc Natl Acad Sci USA. (2012) 109:14652–6. doi: 10.1073/pnas.1212056109, 22908288 PMC3437897

[14] McPherson JG Chen A Ellis MD Yao J Heckman CJ Dewald JPA. Progressive recruitment of contralesional cortico-reticulospinal pathways drives motor impairment post stroke. J Physiol. (2018) 596:1211–25. doi: 10.1113/JP274968, 29457651 PMC5878212

[15] Stinear CM Byblow WD Ackerley SJ Smith MC Borges VM Barber PA. PREP2: a biomarker-based algorithm for predicting upper limb function after stroke. Ann Clin Transl Neurol. (2017) 4:811–20. doi: 10.1002/acn3.488, 29159193 PMC5682112

[16] Byblow WD Stinear CM Barber PA Petoe MA Ackerley SJ. Proportional recovery after stroke depends on corticomotor integrity. Ann Neurol. (2015) 78:848–59. doi: 10.1002/ana.24472, 26150318

[17] Fugl-Meyer AR Jääskö L Leyman I Olsson S Steglind S. The post-stroke hemiplegic patient. 1. A method for evaluation of physical performance. Scand J Rehabil Med. (1975) 7:13–31. doi: 10.2340/1650197771331, 1135616

[18] Wolf SL Catlin PA Ellis M Archer AL Morgan B Piacentino A. Assessing Wolf Motor function test as outcome measure for research in patients after stroke. Stroke. (2001) 32:1635–9. doi: 10.1161/01.STR.32.7.1635, 11441212

[19] Porter BA Khodaparast N Fayyaz T Cheung RJ Ahmed SS Vrana WA . Repeatedly pairing vagus nerve stimulation with a movement reorganizes primary motor cortex. Cereb Cortex. (2012) 22:2365–74. doi: 10.1093/cercor/bhr316, 22079923

[20] Khodaparast N Hays SA Sloan AM Fayyaz T Hulsey DR Rennaker RL . Vagus nerve stimulation during rehabilitative training improves forelimb strength following ischemic stroke. Neurobiol Dis. (2013) 60:80–8. doi: 10.1016/j.nbd.2013.08.002 23954448

[21] Khodaparast N Hays SA Sloan AM Hulsey DR Ruiz A Pantoja M . Vagus nerve stimulation delivered during motor rehabilitation improves recovery in a rat model of stroke. Neurorehabil Neural Repair. (2014) 28:698–706. doi: 10.1177/1545968314521006 24553102 PMC4134702

[22] Hays SA Khodaparast N Ruiz A Sloan AM Hulsey DR Rennaker RL . The timing and amount of vagus nerve stimulation during rehabilitative training affect poststroke recovery of forelimb strength. Neuroreport. (2014) 25:676–82. doi: 10.1097/WNR.0000000000000154, 24818637 PMC4039714

[23] Hays SA Khodaparast N Hulsey DR Ruiz A Sloan AM Rennaker RL . Vagus nerve stimulation during rehabilitative training improves functional recovery after intracerebral hemorrhage. Stroke. (2014) 45:3097–100. doi: 10.1161/STROKEAHA.114.006654, 25147331 PMC4175144

[24] Khodaparast N Kilgard MP Casavant R Ruiz A Qureshi I Ganzer PD . Vagus nerve stimulation during rehabilitative training improves forelimb recovery after chronic ischemic stroke in rats. Neurorehabil Neural Repair. (2016) 30:676–84. doi: 10.1177/1545968315616494, 26542082 PMC4854844

[25] Hays SA Ruiz A Bethea T Khodaparast N Carmel JB Rennaker RL . Vagus nerve stimulation during rehabilitative training enhances recovery of forelimb function after ischemic stroke in aged rats. Neurobiol Aging. (2016) 43:111–8. doi: 10.1016/j.neurobiolaging.2016.03.030, 27255820 PMC5206764

[26] Meyers EC Solorzano BR James J Ganzer PD Lai ES Rennaker RL . Vagus nerve stimulation enhances stable plasticity and generalization of stroke recovery. Stroke. (2018) 49:710–7. doi: 10.1161/STROKEAHA.117.019202, 29371435 PMC6454573

[27] Morrison RA Hulsey DR Adcock KS Rennaker RL Kilgard MP Hays SA. Vagus nerve stimulation intensity influences motor cortex plasticity. Brain Stimul. (2019) 12:256–62. doi: 10.1016/j.brs.2018.10.017, 30409712 PMC6347516

[28] Hulsey DR Hays SA Khodaparast N Ruiz A Das P Rennaker RL . Reorganization of motor cortex by vagus nerve stimulation requires cholinergic innervation. Brain Stimul. (2016) 9:174–81. doi: 10.1016/j.brs.2015.12.007, 26822960 PMC4789078

[29] Hulsey DR Riley JR Loerwald KW Rennaker RL Kilgard MP Hays SA. Parametric characterization of neural activity in the locus coeruleus in response to vagus nerve stimulation. Exp Neurol. (2017) 289:21–30. doi: 10.1016/j.expneurol.2016.12.005, 27988257 PMC5297969

[30] Hulsey DR Shedd CM Sarker SF Kilgard MP Hays SA. Norepinephrine and serotonin are required for vagus nerve stimulation directed cortical plasticity. Exp Neurol. (2019) 320:112975. doi: 10.1016/j.expneurol.2019.112975, 31181199 PMC6708444

[31] Tseng CT Gaulding SJ Dancel CLE Thorn CA. Local activation of α2 adrenergic receptors is required for vagus nerve stimulation induced motor cortical plasticity. Sci Rep. (2021) 11:21645. doi: 10.1038/s41598-021-00976-2, 34737352 PMC8568982

[32] Bowles S Hickman J Peng X Williamson WR Huang R Washington K . Vagus nerve stimulation drives selective circuit modulation through cholinergic reinforcement. Neuron. (2022) 110:2867–2885.e7. doi: 10.1016/j.neuron.2022.06.017, 35858623 PMC10212211

[33] Dickie DA Kimberley TJ Pierce D Engineer N Tarver WB Dawson J. An exploratory study of predictors of response to vagus nerve stimulation paired with upper-limb rehabilitation after ischemic stroke. Sci Rep. (2019) 9:15902. doi: 10.1038/s41598-019-52092-x, 31685853 PMC6828969

[34] Dawson J Engineer ND Prudente CN Pierce D Francisco G Yozbatiran N . Vagus nerve stimulation paired with upper-limb rehabilitation after stroke: one-year follow-up. Neurorehabil Neural Repair. (2020) 34:609–15. doi: 10.1177/1545968320924361, 32476617

[35] Dawson J Engineer ND Cramer SC Wolf SL Dixit A Alexander J . Vagus nerve stimulation paired with rehabilitation for upper limb motor impairment and function after chronic ischemic stroke: subgroup analysis of the randomized, blinded, pivotal, VNS-REHAB device trial. Neurorehabil Neural Repair. (2023) 37:367–73. doi: 10.1177/15459683221129274, 36226541 PMC10097830

[36] Francisco GE Engineer ND Dawson J Kimberley TJ Cramer SC Prudente CN . Vagus nerve stimulation paired with upper-limb rehabilitation after stroke: 2- and 3-year follow-up from the pilot study. Arch Phys Med Rehabil. (2023) 104:1180–7. doi: 10.1016/j.apmr.2023.02.012, 37001842

[37] Kimberley TJ Cramer SC Wolf SL Liu C Gochyyev P Dawson J . Long-term outcomes of vagus nerve stimulation paired with upper extremity rehabilitation after stroke. Stroke. (2025) 56:2255–65. doi: 10.1161/STROKEAHA.124.050479 40329913

[38] Beovich A Boose J Patel R Wolf SL. Vagus nerve stimulation paired with rehabilitation for chronic stroke: characterizing responders. J Neurol Phys Ther. (2024) 48:217–23. doi: 10.1097/NPT.0000000000000488, 39028576

[39] Schwarz A Feldman M Le V Dawson J Liu CY Francisco GE . Association that neuroimaging and clinical measures have with change in arm impairment in a phase 3 stroke recovery trial. Ann Neurol. (2025) 97:709–19. doi: 10.1002/ana.27156, 39676623

[40] Lin S Rodriguez CO Wolf SL. Vagus nerve stimulation paired with upper extremity rehabilitation for chronic ischemic stroke: contribution of dosage parameters. Neurorehabil Neural Repair. (2024) 38:607–15. doi: 10.1177/15459683241258769, 38836606

[41] Vora I Gochyyev P Engineer N Wolf SL Kimberley TJ. Distal versus proximal arm improvement after paired vagus nerve stimulation therapy after chronic stroke. Arch Phys Med Rehabil. (2024) 105:1709–17. doi: 10.1016/j.apmr.2024.05.018, 38815953 PMC11374485

[42] Kumar P. Hemiplegic shoulder pain in people with stroke: present and the future. Pain Manag. (2019) 9:107–10. doi: 10.2217/pmt-2018-0075, 30681020

[43] Kalichman L Ratmansky M. Underlying pathology and associated factors of hemiplegic shoulder pain. Am J Phys Med Rehabil. (2011) 90:768–80. doi: 10.1097/PHM.0b013e318214e976, 21430513

[44] Gutkowska O Martynkiewicz J Urban M Gosk J. Brachial plexus injury after shoulder dislocation: a literature review. Neurosurg Rev. (2020) 43:407–23. doi: 10.1007/s10143-018-1001-x, 29961154 PMC7186242

[45] O’Shea K Feinberg JH Wolfe SW. Imaging and electrodiagnostic work-up of acute adult brachial plexus injuries. J Hand Surg Eur. (2011) 36:747–59. doi: 10.1177/1753193411422313, 21921067

[46] Davidson EJ Tan ET Pedrick EG Sneag DB. Brachial plexus magnetic resonance neurography: technical challenges and solutions. Investig Radiol. (2023) 58:14–27. doi: 10.1097/RLI.0000000000000906, 35926072

[47] Hoffmann TC Glasziou PP Boutron I Milne R Perera R Moher D . Better reporting of interventions: template for intervention description and replication (TIDieR) checklist and guide. BMJ. (2014) 348:g1687. doi: 10.1136/bmj.g1687, 24609605

[48] Lang CE Lohse KR Birkenmeier RL. Dose and timing in neurorehabilitation: prescribing motor therapy after stroke. Curr Opin Neurol. (2015) 28:549–55. doi: 10.1097/WCO.0000000000000256, 26402404 PMC4643742

